# Laying the Foundation of Medical Professionalism among Pre-clinical Students: Importance of Reflection

**DOI:** 10.15694/mep.2019.000103.2

**Published:** 2020-02-12

**Authors:** Prerna Agarwal, Alka Rawekar

**Affiliations:** Datta Meghe Institute of Medical Sciences

**Keywords:** medical professionalism, unprofessionalism, early clinical exposure, reflection, OSCE, pre-clinical students.

## Abstract

This article was migrated. The article was marked as recommended.

***Introduction:*** The escalating problem of unprofessionalism calls for teaching medical professionalism in a manner that should lead to deeper learning. Early clinical exposure (ECE) to an Intensive Care Unit (ICU) presents the issues pertaining to medical professionalism to the students in a more explicit and emotionally challenging manner. And reflection note writing evokes the critical process of thought and analysis required for learning. We conducted the present study to sensitize the pre-clinical students towards medical professionalism using these two tools, ECE and Reflection.

***Methods:*** Two hundred students of 1^st^ MBBS were given an Objective Structured Clinical Examinations (OSCE). The students were then taken for ECE to an ICU. There, the students observed different ongoing activities and critical patients, a doctor discussed some cases with them, and they also interacted with the relatives of patients admitted in the ICU. Thereafter, students wrote a ‘reflection’ note describing what did you see? so what? and now what? Students were again given an OSCE, similar to the one given before the ECE, for assessing any change in their professional behaviour.Analysis of reflection notes was done thematically and of OSCE scores using paired t-test (p<0.05).

***Results:*** The analysis of reflection notes revealed the budding of different elements of professionalism among the students. Post-visit OSCE scores also showed significant improvement.

***Conclusion:*** Incorporation of reflection note writing along with ECE is helpful in laying the foundation of medical professionalism among pre-clinical students.

## Introduction

Medical field is increasingly becoming plagued with unprofessionalism (Leape, 2006; Johnson C, 2009; Bradley *et al*., 2015; Tricco *et al*., 2018). It points out the failure of present medical curriculum in instilling medical professionalism (Swick, 2000; Brennan *et al*., 2002; Indian medical Council Regulations, 2002; Passi *et al*., 2010; Hafferty *et al*., 2012; Riley and Kumar, 2012; Jha *et al*., 2014) among its students.

**Figure 1.**
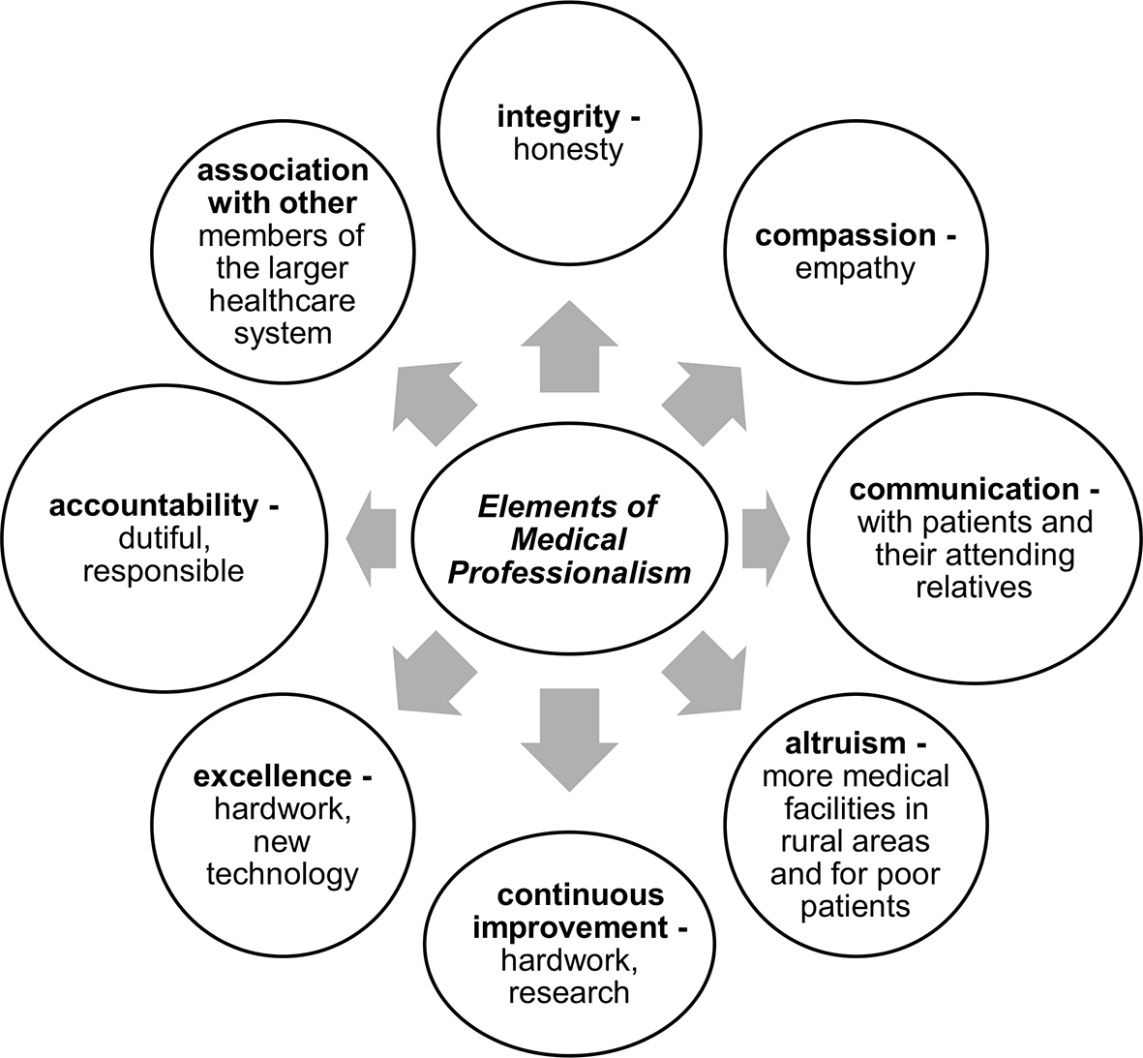
Elements of Medical Professionalism

Teachings regarding medical professionalism usually involve discussions with teachers, seniors, colleagues, role plays, imitations from observation, etc., when the students step into the clinical phase. The clinical exposure of the undergraduate students is also limited to mostly out-patient clinics and wards. Actual professional challenges are faced by them only during their internship and practice as independent doctors. Here, unless the individual’s learning about professionalism so far, has been well directed and well thought of, far beyond hit and trial, the chances of unprofessionalism creeping in become very high. Therefore, there is a pressing need to teach medical professionalism (figure 1) to the students in a manner that leads to deeper learning, i.e. a manner which will provide them an opportunity to observe the profession closely, analyze it critically (reflect on it), and form appropriate behavioral and attitudinal responses; and all this should have early beginnings so that the impressions thereby formed are profound, and professional attitude and behavior become naturalized in due course.

The tools of early clinical exposure (ECE) (Benbassat and Schiffman, 1976; Ali M *et al*., 1977; Johnson and Scott, 1998; McLean 2004; Lie *et al.,* 2006; Basak *et al.,* 2009; *Dornan et al.,* 2009; Helmich *et al*., 2011; Ali K *et al.*, 2018) and reflection (Charon, 2001; Sandars, 2009; Hargreaves, 2016) have been variously used to enhance learning: teaching clinical methods, case base learning, sensitizing students towards patient care, helping them develop their self-identity, motivating them, etc.We believe that these two tools, when used in conjunction, can be used to inculcate the elements of professionalism among pre-clinical students, who are the future doctors. While early clinical exposure will present the conundrums of medical professionalism to the students, reflection note writing will be instrumental in evoking the critical thought and analysis required for addressing them, thereby leading to budding of the elements of medical professionalism among them. For the purpose of clinical exposure, an intensive care unit (ICU), will be an appropriate setting (Qutub, 2000) to observe and understand professional callings closely. In an ICU, the critical patients strive for life and it poses great professional challenges.Further, to reaffirm the impact of the ECE and reflection on professional behaviour in an objective manner, an Objective Structured Clinical Examination (OSCE) may be used (Davis, 2003; Turner and Dankoski, 2008; Brannick, Erol-Korkmaz and Prewett, 2011; Patrício *et al.,* 2013; Falcone, Claxton and Marshall, 2014).

The collective role of ECE and reflection in teaching medical professionalism to pre-clinical students has not been explored. And the pre-clinical students are usually not given exposure to an ICU. It is against this background that we designed and carried out our study, which makes it relevant, novel, and valid. The aim of our study was to sensitize the pre-clinical students towards medical professionalism using these two tools.

## Methods

The empirical research involved 200 students of 1^st^ MBBS, batch 2017-2018. Clearance from Institutional Ethics Committee was obtained (Ref. No. DMIMS(DU)/IEC/2017-18/6792) and a written informed consent of the students was taken.

### Research design

Our study was a mixed methods experimental research. The experiment included reflection note writing, ECE being a regular part of the curriculum for 1^st^ MBBS in the college. There were both qualitative and quantitative components: qualitative component included analysis of reflection notes, using a post-course design, and quantitative component included assessment of OSCE results and feedback; OSCE was incorporated as before and after design.

### Method (figure 2)

An objective structured clinical examination (OSCE) was given to all the students. The OSCE included 3 stations where the students had to perform different components of clinical examination on subjects. The subjects were healthy people from among the staff of the college who had volunteered to be subjects for the same. Each student spent 5 minutes at a station. Among other steps of the clinical examination proper, the students were evaluated for their professional behavior towards the subject and their communication with him/her: 1) greeting the subject, 2) asking his/her name, age, occupation, residence, chief complaints 3) explaining the procedure of performing the examination to the subject, 4) reassuring him/her, 5) taking his/her consent to perform the examination on him/her, 6) exposing the body part required for examination in a gentle and dignified way, 7) being gentle in examination, 8) covering back the exposed part after examination, 9) informing the subject about the completion of examination and its result, and 10) thanking the subject for his/her cooperation. The evaluation for each step of OSCE, including those assessing professionalism, was done by awarding marks from 0 to 1. One (1) mark was awarded when the response of the student was satisfactory or correct and zero (0) when it was incorrect. If the response was less than satisfactory, but not incorrect, the student was awarded less than 1 but more than 0 mark.

For, the purpose of early clinical exposure, the students were taken to an intensive care unit. The students were given a brief introduction as to what an intensive care unit is. They were also told what a reflection/reflection note is and were asked to write and submit the same after the visit. To help them write it, the students were given handouts carrying clues about writing reflection: *what did you see* (what was your observation)? *so what* (what were your feelings and thoughts about it)? and *now what* (what do you intend to do about it in future)?

Thereafter, the students were taken for visiting an ICU in medicine department of hospital attached to the medical college. The students were divided into three batches. Each batch was taken for visit on a separate day. The students were further subdivided into groups of 10-12 students. Only one group went inside the ICU at a time and the other groups interacted with the relatives of the patients admitted in the ICU. Each group spent about 30 minutes inside the ICU under the guidance of a doctor, who discussed with them the ICU set-up, few cases/patients admitted there, and also answered their queries. During their interaction with the relatives of patients, students enquired about the problems they were facing regarding treatment of their patient and their stay in hospital. After the visit, the students were given time to discuss the visit among themselves and with the teacher. The students then wrote reflection notes and submitted them. Copies of the reflection notes were kept and the original ones were returned to them. Then, another OSCE, similar to the one given before the ICU visit, was given to the students and their evaluation was done with respect to the same aspects of empathy, communication and professional attitude, as before. Thereafter, a feedback on the visit was collected from the students by means of a validated questionnaire. The questionnaire had 13 items meant to be valued on a five point Likert scale, ranging from strongly agreeing with an item to strongly disagreeing with it. 7 of these items were related to the identification of the elements of medical professionalism by the students.

**Figure 2.**
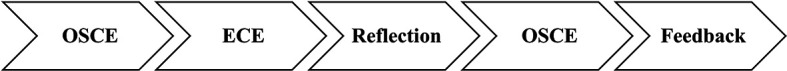
Our method

The qualitative data of reflection notes was analyzed thematically. All the points mentioned by the students were taken into consideration, coded and tabulated by both the authors, separately. The authors then exchanged notes and discussed the themes, coding and interpretations for ensuring exhaustive study of the reflection notes and for cross-checking the results. For analysis of OSCE results, only the scores assessing professionalism were taken into consideration. A paired t- test with p<0.05 as significance level was used. Feedback was also analyzed quantitatively by calculating percentage of students agreeing with a particular value of an item. Thematic analysis was done using QDA Miner Lite 2.0.5 and quantitative analysis using Microsoft Excel Professional 2015.

## Results/Analysis

The 200 students included 92 females and 108 males (table 2).

### Analysis of ICU visit Reflection notes

The reflection notes revealed the dynamics of perception and attitude of the students as they were remodeled by the clinical exposure and experience. The reflection notes were scrutinized under three domains: what did you see? so what? and now what? Several themes emerged each with its own set of relevant codes (table 1). Analysis of each of these themes reflected the budding of different elements of professionalism (figure 1) among the students. Cited below are a few exemplars from the reflection notes which are suggestive of the inculcation of these different elements.

Exemplar 1*: “when we entered the ICU and when I saw the patients, I got to know what must be their mental condition: nothing but painful and helpless. But for this how a doctor takes standing is something that can never be neglected by me.”*

- reveals development of empathy for patients, and of a sense of responsibility.

Exemplar 2: *“.. after all this I realized that to become a doctor is not an easy task, it requires a lot of hard work... in starting, I was taking studies very lightly, but when I saw patients in ICU, I realized we are the future doctors who would deal with patients’ lives. And before all this we should acquire all knowledge ...”*

- reveals realization of importance of hard work for continuous improvement in knowledge and skill, and willingness for striving for excellence.

Exemplar 3: *“The family was in agony and we could see their impatience and helplessness. For them we were all doctors. So, the patient’s wife asked me if he was out of danger. I felt very helpless. At the same time, I understood what this white coat signifies.”*

- reveals development of empathy for relatives of patients and of sense of accountability.

Exemplar 4: *“... and just knowledge is not enough. My body language, my words, what I say in front of relatives of my patients, who believe that he will be well as he has come to me, the way I talk, I dress and my overall behavior with staff also matters. And henceforth I need to inculcate all these things in my behavior and most importantly study hard everything thoroughly.”*

- reveals realization of importance of having good communication skills.

Exemplar 5: *“... After coming outside, I saw another battle of doctors: one of the relatives was so firm in his belief that he was debating with the doctor. But she (doctor) was trying to convince him that they are trying their best to save the patient. But still he was not able to understand.”*

- reveals development of empathy for doctors (other health professionals) and realization of importance of having good communication skills.

Exemplar 6: *“... One of the things that I noticed was the way doctor interacted with the patient and staff. I am glad that I had such a positive experience. I want to be a good doctor, so it is important for me to stay connected with patient...”*

- reveals realization of importance of working in association with other health professionals, and that of need of developing good communication skills.

Exemplar 7: *“What I felt is we should help them at least emotionally. And if possible financially. As we waste a lot of money on other things which are sometimes not useful for us. Instead of that we should help them. This is the most valuable work (helping others emotionally and financially, if possible). In rural hospitals, we can serve food for their relatives which can help them to a certain extent.”*

- reveals a sense of social justice and altruism being developed.

Exemplar 8: *“... just stay honest towards the profession and work hard for your patients.”*

- reveals inculcation of sense of integrity.

Exemplar 9: *“The doctor-patient relationship is the foundation of medical ethics. Patients, the innocent problem holders, come up to doctors for all sorts of problems, be it physical, mental or social. They expect doctors to give solution to every kind of problems. And so, it is our duty to stand up to their mark.”*

- reveals a sense of accountability and integrity.

**Table 1.** Analysis of Reflection Notes of Pre-clinical students after ICU visit

	**Experience of ICU and interaction with relatives of patients**				
	*Most of the students had not been to an ICU before. The students wore cap, mask and shoe covers for going inside ICU. Inside ICU, there were critically ill patients. There was cleanliness and discipline. The silence was broken by sounds of equipment and patients’ cries of agony. There was more staff in ICU than number of patients. Various devices and equipment were attached to the patients for monitoring their condition and treatment. Doctors were examining patients and communicating with ICU staff, including other doctors and nurses. All ICU staff was carefully tending to the patients. A doctor discussed cases of some critical patients admitted there, with the students and answered their queries. Doctors apprised the relatives of patients about their condition and reassured them. There was a confrontation between relatives of a patient and a doctor. One patient’s condition deteriorated. Despite best resuscitation efforts, the patient passed away. The doctor informed his relatives about the same. Students interacted with the relatives of patients admitted waiting outside ICU. There was an initial hesitation but following an exemplar demonstration by the teacher, they asked the relatives about the condition of their patient and about the problems they faced. The relatives treated the students with respect and told them about their problems: monetary constraints, accommodation, food, not being allowed to meet their patient often, not being more informed about the condition of their patient, having to come from far off rural places, unsuccessful diagnosis and treatment at some clinics and hospitals, etc.*				
	**Perception of students before ICU visit**	**What did you see?**	**So what?**	**Now what?**	***Elements of Professionalism reflected***
	**Theme 1- Patients**				
	**Subtheme** - What does it mean to be a patient?				
**Codes**	Life of patient is in a doctor’s hands	Critically ill patients fighting for life	Patients are in a miserable state, they look up to doctors	Treat patients, serve patients	*Empathy for patients, sense of service and responsibility*
	**Subtheme**- Challenges before a patient				
**Codes**	*No mention*	Connected to numerous medical equipment	Suffering due to disease and its treatment	Provide more facilities and comprehensive services to patients, use updated treatment, more service in rural area, better communication and treat patients with respect and care	*Empathy for patients, importance of communication, strive for improving knowledge and skill*
	**Theme 2** - Relatives of patients				
	**Subtheme**- Role of relatives of patients				
**Codes**	*No mention*	Waiting outside ICU, communicating with doctor, cooperating with students, attaching their hope to doctor	Are in miserable condition, are more aware, have faith in doctor, have respect for medical profession	Better communication and treat relatives of patients with respect and care, listen to them	*Empathy for relatives of patients, importance of communication*
	**Subtheme**- Challenges faced by relatives of patients				
**Codes**	*No mention*	Not able to meet their patient often, not informed regularly about patient’s condition, no proper place to stay, confrontation with a doctor, financial constraints	Deplorable condition, poor facilities, do not trust doctors blindly, we (students) waste money that could be put to better use	Better communication with relatives, allow them to meet patient, provide more facilities, free medical service in rural areas and to the poor	*Empathy for relatives of patients, altruism, importance of communication, sense of social justice*
	**Theme 3** - Doctors				
	**Subtheme** - What does it mean to be a doctor?				
**Codes**	Impressed by the white coat that doctors wear, doctors are respected in society, clueless of what exactly is the role of doctor	Treating patients, communicating with other doctors, nurses, relatives of patients, teaching medical students	Work hard, serve patients, patients and relatives have faith in them	More respect for doctors, be a good doctor, work hard in studies and career, cooperate with colleagues	*Empathy for doctors, strive for improving knowledge and skill, cooperation with other members of profession*
	**Subtheme** - Challenges before a doctor				
**Codes**	Clinical experience is required	Very busy and on toes, a confrontation between a doctor and a patient’s relatives, a patient passed away despite resuscitation efforts	Doctors work hard, are in stress, are responsible for patient’s well- being, less faith in them nowadays, better communication with relatives- should treat patients and relatives more empathetically	Work hard, better communication, treat patients and relatives with empathy, update knowledge and skill	*Empathy for doctors, importance of knowledge and skill, excellence, sense of accountability and responsibility, importance of communication*
	**Theme 4** - Doctor-patient relationship and that between doctor and patient’s relatives				
	**Subtheme** - Understanding the relationship between a doctor and the patients and their relatives				
**Codes**	*No mention*	Doctor treating patients, doctor communicating with relatives of patients, confrontation between doctor and patient’s relatives, doctor informing relatives about patient’s demise, relatives attaching hopes to doctor	A doctor-patient relationship exists, doctors should treat patients and their relatives more empathetically, good communication between doctor and relatives is must, lack of complete faith in doctor, doctor is next to god for patients and their relatives	Treat patients and their relatives in a better way, listen carefully to patients and their relatives, develop good communication skills	*Importance of professional behavior, importance of doctor-patient relationship, importance of communication*
	**Theme 5** - Medical studies				
	**Subtheme** - Challenges of medical studies?				
**Codes**	A new experience, theoretical, decreased enthusiasm over time	Case discussion with doctor, medical equipment	Application of theory in clinical scenario, realized importance of studying theory, difficult course	Not neglect theory, acquire more knowledge and keep it updated, be involved in research, will work hard	*Be a life-long learner, be competent, strive for excellence*
	**Theme 6** - Medical Profession				
	**Subtheme** - Perception of medical profession				
**Codes**	Honorable and interesting profession	ICU set up, patients, doctors working in ICU, interaction with relatives of patients	Difficult and painful profession, interesting, a doctor is important for society, requires skill, hard work and practice, less faith in doctors nowadays, all hard-work worthwhile, requires ethical practice	Develop professionalism, work hard, be honest with profession, develop skill and be competence	*Integrity, commitment, importance of communication, sense of responsibility and accountability, be competent, strive for excellence*
	**Theme 7-** Self identity				
	**Subtheme** - Relating self with the professional field				
**Codes**	Becoming a doctor will be dream come true, decreased enthusiasm over time	*the whole clinical experience*	Not seen anything like this before, saw what the profession is all about, thrilling experience, realized responsibility, felt emotional- helpless, shocked, felt connected with the profession for the first time, eagerly waiting to treat patients by own self, more respect for profession	Be a good doctor, work hard, be determined, improve personality, develop professionalism, be emotionally strong, create own identity,	*Dedication, skillfulness, sense of responsibility, strive for excellence*
	**Subtheme** - Relating self with life in general				
**Codes**	*No mention*	Critical patients, problems faced by relatives of patients, doctors working hard	Saw reality of life, felt life is fragile and precious, felt thankful for life and towards parents, realization of responsibility towards poor and needy	Become a good doctor, provide more free services in rural areas, justify the faith entrusted and respect given	*Empathy, ethics, altruism, sense of responsibility*

### Analysis of OSCE performance

Table 2 summarizes the OSCE scores of students before and after the ICU visit. A significant improvement was seen in the performance of students in the OSCE given after the ICU visit.

**Table 2.** OSCE Scores before and after the visit to ICU

	OSCE Mean Score %^a^		paired t-test P < 0.05
	**Before visit**	**After visit**	
**Females**	62.98 (12.22)	69.33 (11.36)	P < 0.001
**Males**	65.58 (11.99)	68.08 (11.77)	P = 0.047
**Total**	64.39 (12.14)	68.65 (11.58)	P < 0.001


^a^ parentheses include standard deviation, S.D.

### Analysis of feedback

Most of the students strongly agreed with the positive influence of ICU visit on various aspects of their medical professional learning (Supplementary file 1). The students either agreed or strongly agreed that seeing critically ill patients aroused their interest in the profession (89.0%), that the agony of relatives for their patients taught them to look at patients sympathetically (91.5%) and that they now had better understanding of importance of communication skills (91.5%). The students also agreed or strongly agreed that the experience motivated them to learn more (95.0%). Most of them agreed or strongly agreed that the experience changed their perception of medical field (82.5%), that they became more sensitive towards their profession (84.5 %), and that they found that their professional attitude has changed after the visit (77.5%). Also, the experience was rated as being quite relevant to pre-clinical phase (88.5%) by the students and found to be helpful in enhancing academic learning (95.0%). These findings suggest that the students were indeed able to identify the different elements of professionalism with the help of the ICU visit and their reflection on it.

## Discussion

The Experiential Learning Theory given by Kolb (Kolb, 1984), states that “learning is the process whereby knowledge is created through the transformation of experience”. There are two processes that are integral to the ‘transformation of experience’- ‘reflection’ on the experience to assimilate information from it and ‘abstract conceptualization’ involving critical comprehension of the events, thereby forming some hypotheses for the observations and an intent to bring that understanding into practice. Without reflection and conceptualization from it, learning cannot take place and the experience loses its meaning. We based our study on this concept.

**Figure 3.**
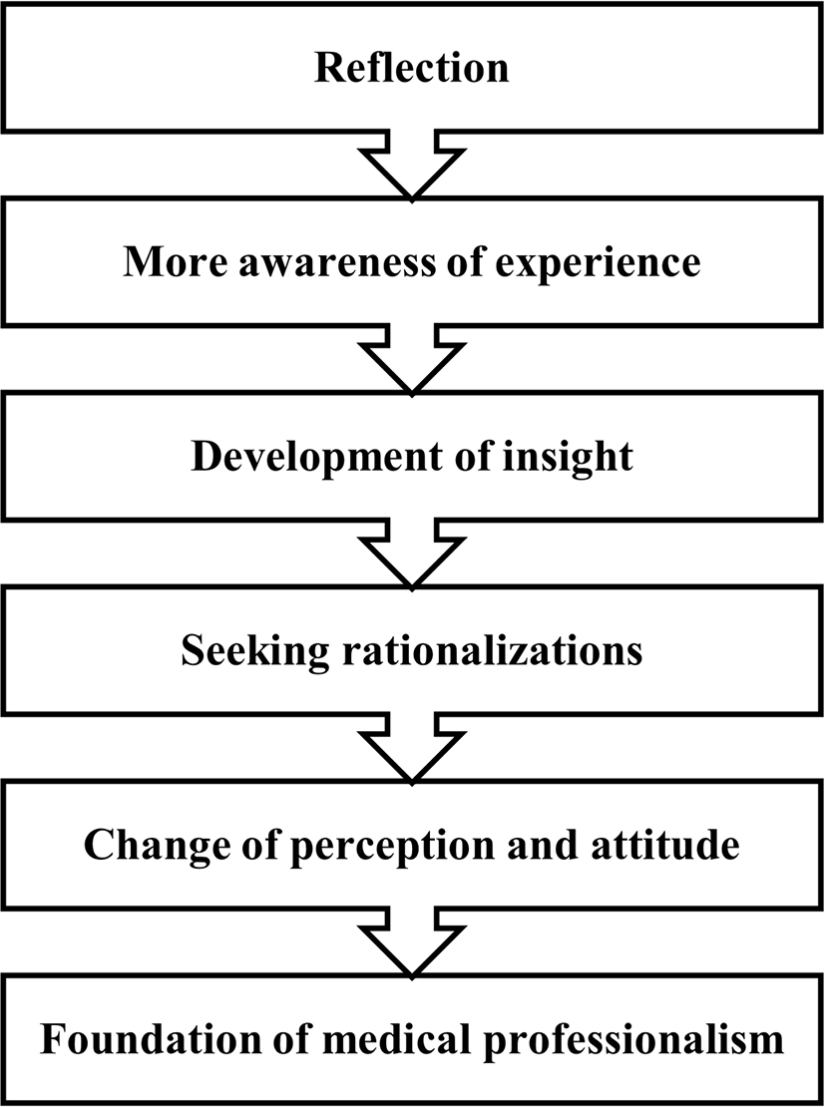
How reflection inculcates medical professionalism

Early clinical exposure lets the pre-clinical and para clinical students become involved in their future work, i.e. clinical setup, at an early stage. Observing the clinical set up, its activities, interaction with patients and doctors, discussions, etc. provide myriads of learning opportunities to the students. One of the earliest published articles on early clinical exposure dates back to 1970s (Benbassat and Schiffman, 1976; Ali M *et al*., 1977) that brought out its benefit in improving academic learning. ECE rekindles the students’ interest in medical sciences, helps them identify their role as a student and as a future doctor (Johnson and Scott, 1998). Over years, other benefits of ECE were revealed and it has been effectively used to teach communication, time management, cultural issues, identity formation, professionalism and self-appraisal as well (Lie *et al.,* 2006; McLean, 2004; Basak *et al.,* 2009; Dornan *et al.,* 2009; Helmich *et al.,* 2011; Ali M *et al.,* 2018). In the present study, the students were taken for a visit of an ICU, the early clinical exposure. The students were then made to write a note on the visit ‘reflecting’ on it. This made them revisit their experience in mind and made them ‘think and analyze it critically’. It made them become more aware of the experience and helped them in developing an insight into it. In turn, this made them seek rationalizations for their thoughts and feelings. Their critical comprehension then reformed their attitude and perception of the experience. And different components of the experience inculcated different elements of medical professionalism among the students (figure 3).

There is no one globally acceptable definition of medical professionalism and the critically relevant attributes of medical professionalism vary (Cruess *et al.,* 2010; Riley and Kumar, 2012; Birden *et al.,* 2013; Jha *et al.,* 2014; Al-Rumayyan *et al.,* 2017) with the socio-economic and cultural environment of work of the professional individual. However, there are some broad elements that can be identified to be characteristic of any good medical professional (Swick, 2000; Passi *et al.,* 2010; Riley and Kumar, 2012; Jha *et al.,* 2014) as depicted in figure 1. The clinical experience introduced the pre-clinical students to these very broadly identified elements of medical professionalism and the critical reflection process helped to lay its foundation in them. Learning from the experience was, therefore, made more concrete with the help of reflection. From being clueless about what the medical profession actually means, the students now began to identify their role as a medical student as well as a future professional doctor.

The significant improvement in the performance of students in OSCE also implies an improvement in their attitude towards the subject on whom the examination was performed. Considering the OSCE result together with students’ reflection notes, it suggests the beginning of development of medical professionalism among them. And thereby, supports our interpretation of the data from their reflection.

Some earlier studies (Pitkälä and Mäntyranta, 2004; Elliott, 2009; Helmich *et al.,* 2012; Wong and Trollope-Kumar, 2014; Borgstrom *et al.,* 2016) have explored reflection as a tool for learning medical professionalism. The results of our study are in conformation with their results. But these studies traced the dynamics of perception and attitude as the students entered the clinical learning stage and maintained a portfolio of the same. While, our study used reflection to teach the same to pre-clinical students during their early clinical exposure. Also, most of these studies involved only few scores of students. Our study analyzed reflection notes of 200 students which makes it very exhaustive. Some of the earlier studies (Pitkälä and Mäntyranta, 2004; Elliott, 2009) were prospective in nature and assessed if reflection helped them be better professionals. But our study was done to sensitize the pre-clinical students towards the same.

In due course of time, as their medical course advances, these students will gain more clinical experience. Then the ‘beginning of medical professionalism’ made in pre-clinical period may guide the future dynamics of their perceptions and attitudes, and may serve to be the foundation of medical professionalism in them.

**Figure 4.**
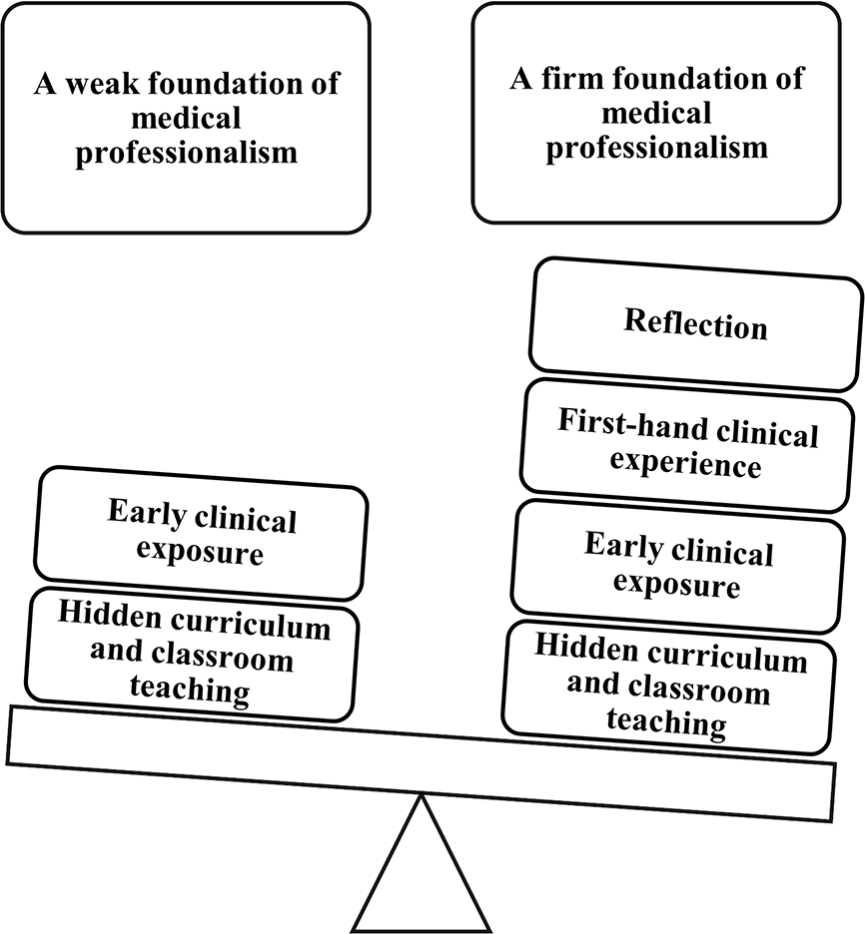
Role of reflection in teaching medical professionalism

Therefore, we may hypothesize that such students, who get clinical exposure and reflect on it in in the pre-clinical period, may become better professionals than those who did not get this opportunity (figure 4). The same may be studied by means of prospective studies.

*Limitations:* An inherent limitation of a qualitative analysis is that it depends on the comprehension of the researchers. But our study analyses the results in a quantitative manner as well, and thereby, tests our qualitative analysis. This gives an edge to our interpretation of the reflection notes and partially overcomes the limitation. And for the same reason, our results are more generalizable than that of a qualitative study alone.

The results of our study may be confounded by the effect of discussion that the students had among themselves and with the teacher after the visit. But learning cannot occur in isolation. It is only appropriate, therefore, to consider it as a part of the process of reflection.

Considering that the students always knew what was the appropriate response, the better performance of the students seems expected. But the purpose of addressing the issue of professionalism is inculcating the same among the students using all available means. The students have to know what is appropriate and that knowledge has to be reinforced repeatedly so that it becomes a part of their spontaneous professional behaviour and attitude.

Another factor that confounds our result, both reflection note writing and OSCE performance, is the student’s tendency to perform better when they know that they are being observed. We would like to bring into consideration here that it’s not a blind study and this bias cannot be done away with; it is an inherent limitation in such studies. However, repetition of any behaviour is essential for learning to occur. The students’ consciously performing better aided in their learning of good professional behaviour and helped in inculcating medical professionalism among the students, which was our aim.

## Conclusion

We conclude that incorporation of reflection note writing with early clinical exposure in the pre-clinical period maybe helpful in inculcating the elements of medical professionalism among the students and may also be helpful in addressing the issue of rising unprofessionalism in medical field.

## Take Home Messages

• Early clinical exposure may be helpful in presenting conundrums of medical professionalism to pre-clinical students.
• Reflection note writing may be helpful in invoking the critical thought and analysis process required for addressing these conundrums.
• Reflection may be helpful in consolidating learning from clinical experience to improve professional behaviour and attitude.
• Early clinical exposure followed by reflection may be helpful in sensitizing pre-clinical students towards medical professionalism.

## Notes On Contributors

**Dr. Prerna Agarwal** (ORCID iD:https://orcid.org/0000-0001-9466-1253) The author obtained her post graduate degree in the year 2013 and has since dedicatedly worked in academics. While teaching undergraduate and post graduate students, she realized that there is immense need of improvising medical education. This research work is her first step in this direction.

**Dr. Alka Rawekar** (ORCID iD: https://orcid.org/0000-0002-1372-6332) The author is a professor of Physiology and is currently positioned as Dean- Allied Health Sciences, JNMC, DMIMS (DU), Wardha. During her academic career of more than 15 years now, she has several articles, both in physiology and medical education, to her credit as she continues to work in the direction of improvising medical education.
