# Supplementary material for: Laying the Foundation of Medical Professionalism among Pre-clinical Students: Importance of Reflection
Source: MedEdPublish (2016). 2020 Feb 12;8:103. Originally published 2019 May 9. [Version 2] doi: 10.15694/mep.2019.000103.2 (PMC10421581; doi:10.15694/mep.2019.000103.2)
Supplement: Peer review history

# Peer review: Reviewer response for version 2

**DOI:** 10.21956/mep.20246.r31399

**Published:** 2020-02-15

**Authors:** Ian Wilson

**Affiliations:** 1 International Medical University

This review has been migrated. The reviewer awarded 4 stars out of 5. This is an interesting paper that promotes early clinical exposure as a method of instilling the beginnings of proessionalism in Year 1 medical students. It is valuable, but you need to be careful aboutoverinterpeting you r results.Does the OSCE as outlined measure some aspect of professionalism. there is a significant debate about whether it is possible to measure professionalism in an OSCE. I may measure some aspect of the approach to a patient. this is only a very small part of professionalism.The reflections are important in meeasuring the impact of the ECE, but it is not clear if it represents a method of teaching prrofessionalism.However sepite my concerns this is an important paper and you should consider extending it.

# Peer review: Reviewer response for version 2

**DOI:** 10.21956/mep.20246.r31397

**Published:** 2020-02-12

**Authors:** Trevor Gibbs

**Affiliations:** 1 AMEE

This review has been migrated. The reviewer awarded 4 stars out of 5. I thank the authors for their insightful response to the reviewers comments and the way that they have restructured their revised paper. I am pleased that the authors have extensively reviewed the limitations of this paper and have striven not to be too dogmatic in the paper's conclusions. As a result of this and I think because the authors have shown due diligence in their second version of the paper, I would now recommend this paper. I feel that the authors have learned from this exercise- it was indeed a very impressive piece of work, and I now look forward to reading further examples from the authors as they build upon developing professionalism in their students- not an easy task and one which requires repetitive learning interventions- but one that remains very important in the curriculum.

# Peer review: Reviewer response for version 2

**DOI:** 10.21956/mep.20246.r31398

**Published:** 2020-02-13

**Authors:** Felix Silwimba

**Affiliations:** 1 University of Lusaka

This review has been migrated. The reviewer awarded 5 stars out of 5. This is an insightful study in medical education for medical students in low in come countries. it can be replicated every where . i recommend this study strongly.

# Peer review: Reviewer response for version 1

**DOI:** 10.21956/mep.18993.r27233

**Published:** 2019-08-10

**Authors:** Ken Masters

**Affiliations:** 1 Sultan Qaboos University

This review has been migrated. The reviewer awarded 3 stars out of 5. An interesting paper dealing with laying the foundations of medical professionalism among pre-clinical students, with an emphasis on using reflection. The authors begin by clearly identifying the problem of unprofessionalism, and then use an exposure session (with a pre- and post-OSCE evaluation) in an ICU as the stimulus for the reflection. The paper does have a few issues that need to be addressed:• The institutional context needs to be more clearly identified.• The OSCE process needs to be laid out in more detail (I see that the authors have described it in response to another reviewer, so, hopefully, Version 2 of the paper will contain this description).• The qualitative data (Exemplar 1 to Exemplar 9) are interesting, and give a good insight into the students’ mindset. When laying out qualitative data, however, it is preferable to identify the theme first, and then to cite examples. In addition, many of the identified themes appear to overlap, and the theme is more of a general description of each exemplar than an actual theme. Part of theming is the process by which separate and succinct themes are identified, and then the data used to support that.• I am not entirely clear what Table 1 is. It appears to be the reflective notes and then analysis of a single student, given as an example, but it could also be information taken from several students. So, I think that, while the data make interesting reading, the authors need to describe a little more explicitly the source of the data in Table 1.• In Table 2, there appear to be errors in the data. The pre mean score for the males and females are given, but the overall mean score is higher than both. As a rough calculation, if the male and female scores are correct, I would estimate that the overall pre mean score should be around 62.77, not 64.39 as given in the table. If this error is merely an error of transcription, then it can be easily corrected; the authors, though, should double-check to ensure that their other associated data (e.g. standard deviations) are not affected. • The data in the supplementary file are actually important data for the paper, so I would recommend that the authors include the table in the body of their paper. • The data in the supplementary file should, however, be presented as raw figures first, and then percentages in parentheses afterwards, not the other way round.• Figure 4 should rather come at the end of the Discussion (before the limitations), and then the Conclusion used to summarise the overall findings of the study.• There are many minor language errors in the paper. For version 2 of the paper, I recommended that the authors proof-read their manuscript very carefully in order to correct these errors.So, I think this is a useful study, but the paper itself does need improvement, and I hope that these suggestions will lead the authors to submit an improved Version 2.

# Peer review: Reviewer response for version 1

**DOI:** 10.21956/mep.18993.r27235

**Published:** 2019-05-24

**Authors:** Virginia Randall

**Affiliations:** 1 Uniformed Services University of the Health Sciences

This review has been migrated. The reviewer awarded 2 stars out of 5. I truly enjoyed reading this paper for the ideas it so well expressed, and for the exemplars it provided of professionalism derived from the students' reflections. Along with other reviewers, I feel the paper did not provide enough details about the OSCEs to fully endorse the paper. I think the paper could be published if rewritten to address this component of the students' evaluation.

# Peer review: Reviewer response for version 1

**DOI:** 10.21956/mep.18993.r27234

**Published:** 2019-05-11

**Authors:** Trevor Gibbs

**Affiliations:** 1 AMEE

This review has been migrated. The reviewer awarded 2 stars out of 5. Although this is an interesting paper to read and does cover an important area, I am not sure that I could be as positive as the previous reviewer.I do think that this paper is an exercise in what constitutes good research and what we can positively take away from the research. I worried with the authors' opening statements regarding the need for teaching- in too many instances teaching doesn't lead to learning and if we are not careful, it only leads to superficial learning. I would agree with the authors regarding their thoughts regarding early clinical exposure and reflective portfolios, both useful learning strategies, but only if used in the correct way. I have no doubt that the students were exposed to a situation- the ICU- that could lead to deep learning, but again, only if used correctly and our results were based on longer term effects.The results from the students were to be expected. Although we are not given the structure of the OSCE- was it a one station or multiple station OSCE and what was the internal structure of the scenario (s)?- the students had been told what was appropriate and I daresay, this was also reflected by their increased scores on the questionnaire. We were not told of how the reflective portfolios were used or scored and what sort of feedback was given, but these are surely important elements to recognise if the student has really learned. At the moment this evaluation lies at only Kirkpatrick 1 and 2- to be as dogmatic as the authors' are in their take home messages I feel that there needs to be a longer-term evaluation to at least level 3 and 4.I am sorry but I am afraid I cannot recommend this paper in its present form, but I do wish the authors well in their future research in this important area.

# Peer review: Reviewer response for version 1

**DOI:** 10.21956/mep.18993.r27232

**Published:** 2019-05-10

**Authors:** Feix Silwimba

**Affiliations:** 1 University of Lusaka

This review has been migrated. The reviewer awarded 5 stars out of 5. I'm encouraged by this study. in my own work I have been grappling with teaching reflection to students of public health as a way of encouraging public health practice professionalism. Unlike clinical medicine education , I cannot find a practicum site similar to ICU experience for students. I find this article relevant to my work.
